# Evaluation of a web-based intervention in patients with chronic human immunodeficiency virus infection: Protocol for a randomized controlled trial

**DOI:** 10.1097/MD.0000000000023683

**Published:** 2020-12-18

**Authors:** Meihua Mao, Gongying Jiang, Qiaofei Jiang

**Affiliations:** Department of infectious disease, Changshan County People's Hospital, Zhejiang, China.

**Keywords:** human immunodeficiency virus, web-based intervention, depression, protocol

## Abstract

**Background:**

The infection of human immunodeficiency virus (HIV) is 1 of the major causes of morbidity and mortality in the world. People with chronic diseases have a higher risk of depression. The HIV people are more likely to suffer from depression. Appropriate psychosocial interventions are effective, but their accessibility is limited by the resources needed for their transmission. Thus, it makes sense to develop more cost-effective alternatives, for instance the web-based intervention (WBI), which may be effective for the well-being and depression. The aim of our program is to explore the effects of a WBI on depressive symptoms and well-being in HIV-infected patients.

**Method:**

It is a randomized controlled experiment to be conducted from February 2021 to July 2021. It was permitted through the Ethics Committee of Changshan County People's Hospital (no.60928376). This study includes 100 HIV patients. Inclusion criteria: (1)18 + years, on effective antiretroviral therapy≥ 1 year before inclusion. Exclusion criteria: patients with severe kidney, liver, lung, and heart diseases. Patients are divided randomly into the study group and control group, each group is assigned 50. The primary results are subjective well-being and depressive symptoms, while the secondary result involves the patients’ satisfaction with life.

**Results:**

The following Table 1 will exhibit the comparison of outcomes between 2 groups.

**Conclusion:**

HIV infected patients can benefit from WBI, which can be utilized as an adjunct to medical treatment.

**Trial registration number:**

researchregistry6215.

## 1 Introduction

The infection of human immunodeficiency virus (HIV) is 1 of the major causes of morbidity rate and mortality in the world, with most of the disease concentrated in Africa.^[1,2]^ More than 75 million people worldwide have been infected with HIV, and there are now approximately 37 million individuals living with the infection.^[3]^ Life expectancy for the HIV people has improved significantly over the past decade, due to the effective antiretroviral therapy.^[4,5]^ It is worth noting that the life expectancy of HIV patients who have been controlled by the antiviral therapy will be similar to the life expectancy of general population. Nevertheless, most of them suffer from the mental disorders.^[6]^ The stigma of becoming HIV positive and the significant losses of self-esteem, friendships, family support, and help, often at a young age around the time of diagnosis can lead to adjustment disorders, which may persist and is likely to have severe consequences.^[7,8]^

The HIV people are more likely to suffer from the depression than the rest of the population.^[9]^ Depression has a debilitating effect on HIV sufferers, and some even think it will accelerate the development of the disease.^[10]^ Therefore, improving well-being and reducing depression are the main treatment targets for HIV infected people. Appropriate psychosocial interventions are effective, but their accessibility is limited by the resources needed for their transmission. Thus, it makes sense to develop more cost-effective alternatives, for instance the web-based intervention (WBI), which may be effective for the well-being and depression.^[11]^ Such interventions are those that use computer technology as the primary or sole medium from which to deliver an intervention. Currently, few studies have examined the WBI in HIV patients. The aim of our program is to explore the effects of a WBI on depressive symptoms and well-being in HIV-infected patients.

## 2 Methods

### 2.1 Study design

It is a randomized controlled experiment to be conducted from February 2021 to July 2021. It was permitted through the Ethics Committee of Changshan County People's Hospital (no.60928376), and this experiment was registered with research registry (researchregistry6215).

### 2.2 Randomization

This study includes 100 HIV patients. A random number is assigned to all the patients through utilizing via using a random number table, and the allocation result is hidden in a random envelope. Patients are divided randomly into the study group and control group, each group is assigned 50 people. Inclusion criteria: (1)18 + years, on effective antiretroviral therapy≥ 1 year before inclusion.

Exclusion criteria: patients with severe kidney, liver, lung, and heart diseases.

### 2.3 The intervention

WBI is designed on the basis of the principles of positive psychology and metacognitive therapy to improve well-being and reduce depression. It includes a total of 14 sessions in a month. For each session, the users are given an email with unique link. Via clicking a link, the users will be guided to a pre-determined websites sequence, which are unique to a particular session. It reads as follows. Day 1 (Introduction): Offer the structure of project, and then build up the treatment credibility. Day 2 (Positive psychology):

(1) List the pleasant activities;
(2) Make the plan of action for enjoyable activities.

Day 3 (HIV and Stress): Practice mindfulness, through focusing on ones breath to clear the patients’ mind and become grounded in the present moment. Day 4 (Self-esteem):

(1) Display of personality advantages;
(2) Identifying the 4 personality traits that are “most resemble me.”

Day 5 (Adapting to the negative experiences): Attributing success to personal, overall and stable characteristics, and failures to situational, specific and temporary characteristics. Day 6 (Dealing with negative events in one's life): Structured GUIDANCE on the expressive writing of negative events to integrate and organize emotions and thoughts. Day 7 (Flow):

(1) Identification of activities that foster flow;
(2) Operational planning of flow activities (namely, where, when and what);
(3) Practicing the mindfulness through paying attention to external cues.

Day 8 (Savoring):

(1) To generate, enhance and then prolong the joyous moments of the past;
(2) To count one's blessings to develop a taste for the positive life experiences.

Day 9 (Metacognitive therapy):

(1) suppression-reverse inhibition experiment to prove the influence of thought control;
(2) Postponement in rumination or worry;
(3) The conduction intentions to postpone worries.

Day 10 (Rumination and worry):

(1) Worry postponement follow-up;
(2) Automatizing postponement of worry.

Day 11 (Thought control):

(1) Instructions on how to observe the thoughts of separation from self;
(2) Review worry postponement task;
(3) The conduction intention to continue to postpone worries.

Day 12 (Detached mindfulness, part one): In the use of imagery (negative) ideas are seen as the clouds floating across sky (namely, it is impossible to control clouds). Day 13 (Detached mindfulness, part 2): Daydreaming technique, in which users practice the transition from being immersed in daydreaming to being the detached observer. Day 14 (Repetition and summary): The summary of the most significant exercises and tasks.

### 2.4 Outcome measures

The primary results are subjective well-being and depressive symptoms, while the secondary result involves the patients’ satisfaction with life.

### 2.5 Statistical Analysis

The analysis of data is carried out with software of SPSS 20.0. Chi-square test is applied for the comparison of descriptive characteristics of patients between the control group and study group. The comparison of the values is conducted by independent samples *t*-test and paired-samples *t*-test. *P* value less than .05 indicates that there is statistical significance.

## 3 Results

The following Table 1 will exhibit the comparison of outcomes between 2 groups.

**Table 1:** The comparison of outcomes between study group and control group.

	Study group (n = 50)	Control group (n = 50)	*P* value
Center for Epidemiological Studies-Depression Scale			
Satisfaction with life scale			
Subjective well-being			
Affect balance			
Depressive symptoms			

## 4 Discussion

Depression is a familiar mental illness, often accompanied by sadness and the loss of interest in things and unable to carry out their daily work.^[12,13]^ The interruption of treatment and HIV infection can lead directly to many psychological problems, as these 2 conditions are often comorbidities.^[14,15]^ The HIV people are more likely to suffer from depression. Nevertheless, depression, which still is neglected in HIV cases, is a dangerous condition that can result in a negative impact not only on adherence to treatment, life quality and social participation, but also on life expectancy and disease progression for people living with HIV.^[16,17]^ Post-discharge intervention is a long-term process of rehabilitation and is critical to improve the patients’ life quality. WBI can encourage the patients to take part in disease self-management and strengthen health education via utilizing video storage and voice messages. Based on our study protocol, further randomized controlled trial is required.

## 5 Conclusion

HIV infected patients can benefit from WBI, which can be utilized as an adjunct to medical treatment.

## Author contributions

Meihua Mao plan the study design and write the manuscript. Gongying Jiang review the protocol. Qiaofei Jiang collect data. All authors approve the submission.

**Conceptualization:** Gongying Jiang.

**Data curation:** Gongying Jiang.

**Funding acquisition:** Meihua Mao.

**Investigation:** Qiaofei Jiang.

**Methodology:** Qiaofei Jiang.

**Writing – original draft:** Meihua Mao.
